# Effect of gene polymorphisms on the mechanical properties of human tendon structures

**DOI:** 10.1186/2193-1801-2-343

**Published:** 2013-07-25

**Authors:** Keitaro Kubo, Hideaki Yata, Naoya Tsunoda

**Affiliations:** Department of Life Science (Sports Sciences), University of Tokyo, Komaba 3-8-1, Meguro-ku, Tokyo, 153-8902 Japan; Sports Science Laboratory, Wako University, Machida, Tokyo, Japan; Faculty of Physical Education, Kokushikan University, Tokyo, Japan

**Keywords:** Ultrasonography, Strain, Stiffness, Collagen

## Abstract

Recent studies showed that polymorphisms in alpha 1 chains of types I (*COL1A1*) and V (*COL5A1*) collagen, growth and differentiation factor 5 (*GDF5*), and matrix metalloproteinase 3 (*MMP3*) genes were associated with injuries in tendons and ligaments (e.g., September et al. (Br J Sports Med 43: 357–365 2009)). In the present study, we aimed to investigate the effects of injury-associated polymorphisms within these four genes on the mechanical properties of human tendon structures *in vivo*. One hundred Japanese males participated in this experiment. The mechanical properties of tendon structures in knee extensors and plantar flexors were measured using ultrasonography. All subjects were genotyped for *COL1A1* rs1800012, *COL5A1* rs12722, *GDF5* rs143383, and *MMP3* rs679620 single nucleotide polymorphisms. For *COL1A1*, all subjects had a GG genotype. For *COL5A1*, maximal tendon elongation and strain of individuals with a CC genotype were significantly greater than individuals with other genotypes (combined TT and CT) for knee extensors, but not for plantar flexors. For *GDF5* and *MMP3*, there were no differences in the mechanical properties of tendon structures in knee extensors and plantar flexors among the three genotypes. The present study demonstrated that subjects with a CC genotype of the *COL5A1* gene had more extensible tendon structures than those of subjects with other genotypes (combined TT and CT) for knee extensors, but not for plantar flexors. The results presented in this study need to be confirmed in a larger cohort of subjects.

## Introduction

Recent studies showed that polymorphisms within alpha 1 chains of types I (*COL1A1*) and V (*COL5A1*) collagen, growth and differentiation factor 5 (*GDF5*), and matrix metalloproteinase 3 (*MMP3*) genes were associated with tendon and/or ligament injuries (Posthumus et al. [Bibr CR25][Bibr CR26][Bibr CR27]; Raleigh et al. 2009; September et al. 2009). On the other hand, the mechanical properties of tendons and ligaments would be expected to be one of the risk factors for these injuries. More recently, Collins et al. (2009) and Brown et al. (2011) demonstrated that the *COL5A1* rs12722 single nucleotide polymorphism was related to range of motion in the lower limb. Furthermore, Kato et al. (2010) suggested that an increase in range of motion due to static stretching was attributable to a change in tendon, not muscle, stiffness. Considering these points, the mechanical properties, such as maximal elongation and stiffness, of tendons and ligaments would be associated with gene polymorphisms mentioned above.

For the last decade, several reports have used ultrasonography to investigate the relationship between tendon properties and performances during stretch-shortening cycle exercises (Kubo et al. 1999,2000,2011; Stafilidis and Arampatzis, 2007). In addition, some previous studies have demonstrated the effects of resistance training on the mechanical properties of human tendons *in vivo* (Kongsgaard et al. 2007; Kubo et al. 2001,2007,2009; Reeves et al. 2003). According to these previous findings, we have no means of enhancing the extensibility of tendon structures, i.e., tendon properties change to be suitable for stretch-shortening cycle exercises, except for bed rest (Kubo et al. 2004; Reeves et al. 2005) and detraining (Kubo et al. 2010). Furthermore, cross-sectional studies demonstrated that tendon structures were more compliant in excellent sprinters compared to inferior sprinters and untrained subjects for knee extensors, but not for plantar flexors (Kubo et al. 2000,2011; Stafilidis and Arampatzis, 2007). Accordingly, it has been assumed that these compliant tendon structures in excellent sprinters are partly determined by genetic factors. In particular, this tendency would be found more clearly in knee extensors than in plantar flexors.

In the present study, we aimed to investigate the effects of single nucleotide polymorphisms within *COL1A1*, *COL5A1*, *GDF5*, and *MMP3* genes previously shown to be associated with tendon and/or ligament injuries (Posthumus et al. [Bibr CR25][Bibr CR26][Bibr CR27]; September et al. 2009) on the mechanical properties of human tendon structures (outer tendon and aponeurosis) *in vivo*. In addition, we also examined whether site-differences in these relationships were found between knee extensors and plantar flexors.

## Methods

### Subjects

One hundred Japanese males (age: 22.0 ± 3.3 yrs, height: 172.6 ± 5.5 cm, body mass: 67.9 ± 10.4 kg, mean ± SD) participated in this experiment. They were undergraduate and graduate students of three universities. When data were collected, subjects were involved in recreational sports activity on average not more than twice per week or 1 hour per week in the past 3 years. None of the subjects reported any current or recent lower limb injuries in the 3 years before testing. Subjects were fully informed of the procedures to be utilized as well as the purpose of this study. Written informed consent was obtained from all subjects. This study was approved by the office of the Department of Sports Sciences, University of Tokyo, and complied with their requirements for human experimentation.

### Elongation and stiffness of tendon structures

Maximal voluntary isometric contraction (MVC) was measured by means of specially designed dynamometers (Applied Office, Tokyo, Japan) for knee extension and plantar flexion, respectively. All measurements were performed on the right lower limb. During each task, subjects exerted isometric torque from zero (relax) to MVC within 5 s. Torque signals were amplified and sampled at 1 kHz using a 16-bit A/D converter (PowerLab/16SP, AD Instruments, Australia). During the knee extension task, the hips and back were held tightly in the seat using adjustable lap belts. The right ankle was firmly attached to the lever arm of the dynamometer with a strap and fixed with the knee joint flexed at an angle of 90 deg (full extension = 0 deg). During the plantar flexion task, subjects lay prone on a test bench and the waist and shoulders were secured by adjustable lap belts and held in position. The ankle joint was set at 90 deg with the knee joint at full extension and the right foot was securely strapped to a footplate connected to the lever arm of the dynamometer.

Elongations in tendon structures (outer tendon and aponeurosis) of knee extensors and plantar flexors were assessed during isometric contractions. An ultrasonic apparatus (SSD-6500, Aloka, Tokyo, Japan) with an electronic linear array probe (7.5-MHz wave frequency with 80 mm scanning length; UST 5047–5, Aloka) was used to obtain longitudinal ultrasonic images of vastus lateralis and medial gastrocnemius muscles by procedures described previously (Kubo et al. 2007,2009). Two measured sites were selected for measurements: at 50% of the distance between the greater trochanter and the lateral epicondyle of the femur for vastus lateralis muscle and at 30% of the distance between the popliteal crease and the centre of the lateral malleolus for medial gastrocnemius muscle. Ultrasonic images were recorded on videotape at 30 Hz and synchronized with recordings of a clock timer for subsequent analysis. The point at which one fascicle was attached to the aponeurosis was visualized on ultrasonic images. The displacement of this point is considered to indicate lengthening of the deep aponeurosis and distal tendon. To correct measurements taken for tendon and aponeurosis elongation, additional measurements were taken under passive conditions (Kubo et al. 2007,2009). For each subject, the displacement of each site obtained from ultrasonic images could be corrected for that attributed to joint rotation alone. In this study, only values corrected for angular rotation were reported. The tendon elongation value (L) was converted to strain by the following equation (Kubo et al. 1999):

Strain (%) = L · TL^-1^ · 100

where TL is the length of the tendon structure at rest. We measured the distance between the measurement site for L and the insertion of the patella and Achilles tendons (confirmed using ultrasonography).

Torque (TQ) measured during isometric contractions was converted to muscle force (Fm) by the following equation (Kubo et al. 2007,2009):

Fm = k · TQ · MA^-1^

where k is the relative contribution of physiological cross-sectional area in each vastus lateralis muscle within knee extensors and medial gastrocnemius muscle within plantar flexors, and MA is the moment arm length in each quadriceps femoris muscles at 90 deg and triceps surae muscle at 90 deg, which was estimated from the limb length of each subject. In this study, Fm and L above 50% of MVC were fitted to a linear regression equation, the slope of which was adopted as stiffness (Kubo et al. 2007,2009).

In a preliminary study, the repeatability of the tendon properties measurement was investigated on 2 separate days with 10 male among all subjects. The coefficient of variation was 5.8% for maximal strain and 6.3% for stiffness.

### DNA extraction and genotyping

Total DNA was isolated from saliva (2 ml) using Orangene DNA (DNA Genotek, Ottawa, Ontario, Canada). Saliva samples were stored at room temperature until total DNA extraction. Genotypes of four polymorphisms {*COL1A1* rs1800012 (G/T), *COL5A1* rs12722 (T/C), *GDF5* rs143383 (T/C), and *MMP3* rs679620 (G/A)} were determined at G&G Science (Fukushima, Japan) by a method that combines PCR and sequence-specific oligonucleotide probes with suspension array technology (Luminex, Austin, Texas, USA). Primers and probes for genotyping are shown in Table 1. Detailed genotyping methodology was described previously (Itoh et al. 2005).

**Table 1:** **PCR primers and probes used for genotyping**

Gene symbol	Polymorphism	Sense primer	Antisense primer	Probe 1	Probe 2
COL1A1 rs1800012	G1245T (intron1)	ATCAgCCgCTCCCATTCTC	AgggAggAgAgAAgggAggTC	CCTCATCCCgCCCCCATTCC	TgCCCAgggAATgTgggCg
COL5A1 rs 12722	C/T (3’ UTR)	gAATCACATgACCTAgCTgCAC	gAgACCTATTCACgAACAggATg	TCTgTCCACACCCACgCgCC	ggCgCATgggTgTggACAgA
gdf5 rd 143383	-/C/T (5‘ UTR)	AgCCTTATACAAgCCTCCTTC	gTgCACCgTCTCCAgTCAg	gAAAggAgAAAgCCgACCgC	TgAAAggAgAAAgCCAACCgC
MMP3 rs679620	A198G (Lys45Glu)	CCTAAAAACTATACTTATTCTgTTAgAAATATCTAg	gATTTTTTTAACAACAggACCACTgTC	gACCTCAAAAAAgATgTgAAACA	gACCTCgAAAAAgATgTgAAAC

### Statistics

Descriptive data are represented as the means ± SD. Any significant differences in measured variables among the three-genotype groups were tested by a one-way ANOVA. When the overall F value was significant, a Tukey’s honest significance post hoc test was used to determine specific differences. The level of significance was set at p<0.05.

## Results

For *COL1A1* rs1800012 (G/T), all subjects had a GG genotype. For *COL5A1* rs12722 (T/C), *GDF5* rs143383 (T/C), and *MMP3* rs679620 (G/A), there were no significant differences in age, height, or body mass between the three genotype groups of each single nucleotide polymorphism (Table 2).

**Table 2:** **Age and physical characteristics of all subjects according to genotypes of polymorphisms**

Mean (SD)				
COL1A1 rs1800012	GG	GT	TT	p value
n	100	0	0	
Age (yr)	22.0 (3.3)	-	-	-
Height (cm)	172.6 (5.5)	-	-	-
Body mass (kg)	67.9 (10.4)	-	-	-
COL5A1 rs12722	TT	CT	CC	p value
n	2	22	76	
Age (yr)	21.0 (2.3)	22.1 (4.0)	22.1 (3.1)	0.894
Height (cm)	172.8 (2.4)	172.4 (4.8)	172.7 (5.8)	0.976
Body mass (kg)	66.1 (6.7)	70.2 (11.1)	67.4 (10.2)	0.771
GDF5 rs 143383	CC	CT	TT	p value
n	8	35	57	
Age (yr)	21.6 (2.6)	21.3 (2.8)	22.5 (3.6)	0.214
Height (cm)	170.7 (8.3)	173.0 (4.8)	172.7 (5.5)	0.573
Body mass (kg)	64.3 (13.4)	65.9 (9.1)	69.4 (10.6)	0.198
MMP3 rs679620	AA	AG	GG	p value
n	10	40	50	
Age (yr)	21.2 (2.1)	21.7 (2.6)	22.4 (3.9)	0.443
Height (cm)	174.6 (6.1)	171.8 (5.0)	173.1 (5.8)	0.339
Body mass (kg)	69.6 (14.1)	66.6 (9.0)	68.5 (10.9)	0.632

For *COL5A1*, the subjects of TT and CT genotypes combined, since the number of subjects with a TT genotype was only two. In both knee extensors and plantar flexors, there were no significant differences (p>0.05) in the MVC values between *COL5A1* (Table 3), *GDF5* (Table 4), and *MMP3* (Table 5) genotype groups. For *COL5A1*, maximal tendon elongation and strain of individuals with a CC genotype were significantly greater than individuals with other genotypes (combined TT and CT) for knee extensors (p=0.012 for maximal elongation, p=0.008 for maximal strain), but not for plantar flexors (both p>0.05) (Table 3). Similarly, the stiffness of individuals with a CC genotype was significantly lower compared to other genotypes (combined TT and CT) in knee extensors only (p=0.013). For *GDF5* (Table 4) and *MMP3* (Table 5), there were no significant differences (p>0.05) in the mechanical properties of tendon structures among the three genotype groups of each single nucleotide polymorphism.

**Table 3:** **Mechanical properties of tendon structures in COL5A1 rs12722 genotype groups**

Mean (SD)				
		**TT + CT**	**CC**	
		**n = 24**	**n = 76**	**p value**
Knee extensors	MVC (Nm)	191 (55)	189 (57)	0.822
	Maximal elongation (mm)	21.1 (5.4)	24.5 (5.4)	0.012
	Maximal strain (%)	6.51 (1.58)	7.61 (1.62)	0.008
	Stiffness (N mm^-1^)	78.2 (18.5)	66.2 (19.3)	0.013
Plantar flexors	MVC (Nm)	129 (29)	126 (25)	0.612
	Maximal elongation (mm)	17.6 (3.6)	18.0 (3.7)	0.631
	Maximal strain (%)	6.39 (1.56)	6.43 (1.33)	0.382
	Stiffness (N mm^-1^)	33.5 (12.4)	35.7 (13.1)	0.493

**Table 4:** **Mechanical properties of tendon structures in GDF5 rs143383 genotype groups**

Mean (SD)					
		**CC**	**CT**	**TT**	
		**n = 8**	**n = 35**	**n = 57**	**p value**
Knee extensors	MVC (Nm)	163 (65)	192 (59)	192 (53)	0.476
	Maximal elongation (mm)	23.0 (6.6)	24.7 (6.3)	23.2 (4.9)	0.333
	Maximal strain (%)	7.39 (2.08)	7.67 (1.90)	7.16 (1.44)	0.294
	Stiffness (N mm^-1^)	62.8 (29.9)	68.7 (18.8)	72.7 (21.8)	0.594
Plantar flexors	MVC (Nm)	119 (34)	219 (28)	127 (24)	0.652
	Maximal elongation (mm)	17.8 (5.2)	18.0 (3.2)	17.9 (3.7)	0.989
	Maximal strain (%)	6.54 (1.76)	6.51 (1.24)	6.35 (1.41)	0.857
	Stiffness (N mm^-1^)	32.2 (11.2)	33.3 (12.4)	36.6 (13.5)	0.424

**Table 5:** **Mechanical properties of tendon structures in MMP3 rs679620**

Mean (SD)					
		**AA**	**AG**	**GG**	
		**n = 10**	**n = 40**	**n = 50**	**p value**
Knee extensors	MVC (Nm)	203 (53)	193 (55)	184 (58)	0.621
	Maximal elongatin (mm)	24.8 (4.5)	23.1 (6.2)	24.0 (5.2)	0.656
	Maximal strain (%)	7.53 (1.32)	7.16 (1.84)	7.49 (1.58)	0.641
	Stiffness (N mm^-1^)	67.8 (119)	73.2 (23.0)	68.6 (21.8)	0.580
Plantar flexors	MVC (Nm)	126 (31)	128 (28)	126 (24)	0.947
	Maximal elongatin (mm)	18.3 (2.8)	17.4 (3.9)	18.3 (3.6)	0.516
	Maximal strain (%)	6.55 (0.98)	6.29 (1.55)	6.51 (1.30)	0.743
	Stiffness (N mm^-1^)	36.8 (17.3)	36.1 (12.5)	34.2 (12.7)	0.752

## Discussion

The main finding of this study was that subjects with a CC genotype of the *COL5A1* gene had more extensible tendon structures compared to subjects with other genotypes (combined TT and CT) for knee extensors, but not for plantar flexors. To our knowledge, this is the first study to demonstrate the relationship between any mechanical properties of tendon structures and a gene polymorphism *in vivo*.

This study suggested the possibility that tendon structures of individuals with a *COL5A1* rs12722 CC genotype were more extensible than individuals with other genotypes (combined TT and CT). A previous study suggested that the *COL5A1* gene was associated with benign joint hypermobility syndrome (Grahame, 1999). More recently, Collins et al. (2009) and Brown et al. (2011) reported that the *COL5A1* rs12722 single nucleotide polymorphism was associated with range of motion in the lower limb. Several researchers have suggested that the major factor contributing to range of motion, i.e., flexibility, is the extensibility of muscles and tendons (Jewell and Wilkie, 1958; Kato et al. 2010; McHugh et al. 1998). Therefore, the present result was supported by the findings of Brown et al. (2011) and Collins et al. (2009). On the other hand, Goncalves-Neto et al. (2002) and Satomi et al. (2008) reported that damaged and pathological tendons contained relatively higher proportion of collagen type III and V, and these alterations were accompanied by a reduction in type I collagen. According to previous findings (Birk, 2001; Roulet et al. 2007), type V collagen expression levels are critical in determining fiber diameter and strength, although type V collagen is a quantitatively minor fibril-forming collagen. In addition, type V collagen gene expression can be, at least in part, determined by polymorphisms within the 3’-UTR of *COL5A1* (Laguette et al. 2011). Therefore, we may say that *COL5A1* gene expression, and by implication type V collagen production, is one of the factors that determine the mechanical properties of human tendon structures.

On the other hand, there were no differences in tendon properties in plantar flexors among the three genotypes of *COL5A1* (Table 3). This implied that the degree of genetic effects on tendon properties is different between knee extensors and plantar flexors. Cross-sectional studies demonstrated that tendon structures were more compliant in excellent sprinters than that in inferior sprinters and untrained subjects for knee extensors, but not for plantar flexors (Kubo et al. 2000,2011; Stafilidis and Arampatzis, 2007). In addition, according to longitudinal studies (e.g., Kubo et al. 2007), we have no training protocol to enhance the extensibility of tendon structures. Considering these points, it has been assumed that these compliant tendon structures for knee extensors in excellent sprinters are partly determined by genetic factors. In addition, our previous study showed that age-associated muscle thickness loss in plantar flexors was less than that in knee extensors (Kubo et al. 2003). The reasons for the differences in the declines in muscle thickness with aging were unclear, but several possibilities exist, i.e., postnatal and genetic factors. In particular, these discrepancies may be due to differences in the daily activity levels between knee extensors and plantar flexors. Indeed, some previous studies indicated that the relative activation level and exerted torque of plantar flexors are higher than those of knee extensors during normal walking (DeVita et al. 1996; Ericson et al. 1986). Considering these points, it seems reasonable to suppose that the mechanical properties of tendon structures for plantar flexors are affected greatly by a postnatal factor.

In previous studies between gene polymorphisms and tendon injuries (Posthumus et al. [Bibr CR25][Bibr CR26][Bibr CR27]; Raleigh et al. 2009; September et al. 2009), South African and Australian and Caucasian populations were investigated. To date, no studies have investigated this theme in Japanese populations. For all gene polymorphisms (*COL1A1*, *COL5A1*, *GDF5*, and *MMP3*), the distribution of each gene polymorphism in the present study was different from previously reported distributions in Caucasian populations (Posthumus et al. [Bibr CR25][Bibr CR26][Bibr CR27]; Raleigh et al. 2009; September et al. 2009). We hypothesized that the genotype of *COL1A1* rs180002 single nucleotide polymorphism, in which more than one study previously reported the relationship between gene polymorphism and injuries (Posthumus et al. [Bibr CR25][Bibr CR26]), is associated with tendon mechanical properties. Unfortunately, the genotype of this gene (rs180002) was the same among subjects in the present study. Genotype distributions of *COL5A1*, *GDF5*, and *MMP3* polymorphisms were, however, similar to the distributions reported in public databases for Japanese populations (http://www.ncbi.nlm.nih.gov/SNP).

In the present study, we must draw the attention to the limitations and assumptions of the methodology followed. Firstly, we measured the tendon elongation at the one point of insertion of a fascicle into the aponeurosis. Two measured sites were selected for measurements: at 50% of the thigh length for vastus lateralis muscle and at 30% of the lower leg length for medial gastrocnemius muscle. Therefore, we may say that these measured sites were relatively same among all subjects. Furthermore, our previous study demonstrated that there was no difference in maximal strain of tendon structures among the proximal, central, and distal sites (Kubo et al. 2002). Therefore, we considered that this point did not affect the main results of this study. Secondly, we must confirm that there was no difference in activity level or loading history between the groups with the different genotypes. The subjects in the present study had engaged in recreational sports activity on average not more than twice per week or 1 hour per week in the past 3 years. In addition, there were no differences in MVC (Tables 3, 4 and 5) and muscle thickness (not showing these data) among the genotype groups. Therefore, we considered that there was no difference in activity level or loading history among the genotype groups. Thirdly, the present study was performed on a small sample size. Nevertheless, the present results showed that tendon structures in knee extensors of subjects with a CC genotype of the *COL5A1* gene were more extensible than those with the other genotypes. In a future study, the results presented in this study need to be confirmed in a larger cohort of subjects.

In conclusion, the present study demonstrated that the *COL5A1* rs12722 genotype, but none of the three other variants investigated, was associated with the mechanical properties of human tendon structures *in vivo*. In knee extensors only, the tendon structures of subjects with a CC genotype of the *COL5A1* gene were more extensible than those with the other genotypes (combined TT and CT). In a future study, these conclusions await additional data for clarification in a larger cohort of subjects. Furthermore, according to predictive genomics DNA profiling for athletic performance, knowledge of genetic suitability in respect to physical function (e.g., speed, endurance) may be useful for the selection of appropriate sporting event (Kambouris et al. 2012). Therefore, it is possible that the extensibility of tendon structures, related to the performances during stretch-shortening cycle exercises, may be predicted by the genotype of the *COL5A1* gene. Further studies are needed to examine whether compliant tendons in excellent sprinters are caused by a genetic factor.
